# Sexuality, rights and personhood: tensions in a transnational world

**DOI:** 10.1186/1472-698X-11-S3-S5

**Published:** 2011-12-16

**Authors:** Dina M Siddiqi

**Affiliations:** 1 Women and Gender Studies Program, Hunter College CUNY, 695 Park Avenue, New York, NY 10065, USA

## Abstract

**Background:**

This article discusses what happens when normative ‘global’ discourses of rights and individuated sexual identity confront the messiness of ‘local’ realities. It considers the tensions that emerge when the relationship between sexual and social identities is not obvious and the implications of such tensions for public health and sexual rights activism. These questions are addressed through debates over the naming of male-to-male sexualities and desires in the context of globalization and the growth of a large NGO (non-governmental organization) sector in urban Bangladesh.

**Methods:**

The material in the paper draws on a research project undertaken in 2008-9 in Dhaka, Bangladesh. A fundamental objective was to produce a contextualized understanding of sexuality in Dhaka city. Methods used included structured interviews, focus group discussions and informal conversations with a range of participants (students, factory workers, public health professionals and sexual minorities). The aim was to generate a conceptual and analytical framework around sexuality and rights rather than to undertake an empirical survey of any one population.

**Results:**

As descriptors, globalized identity categories such as Men who have Sex with Men (MSM), used by public health providers, the state and donors; and gay/lesbian, invoked by human rights activists and transnational NGOs, are too narrow to capture the fluid and highly context-specific ways in which gender and sexually nonconforming persons understand themselves in Bangladesh. Further, class position mediates to a significant degree the reception, appropriation or rejection of transnational categories such as MSM and Lesbian, Gay, Bisexual, Transgender (LGBT). The tension is reflected in the sometimes fraught relations between service providers to MSM, the people they serve and an emerging group who identify as LGBT.

**Conclusion:**

A simple politics of recognition will be inadequate to the task of promoting health and human rights for all; such a strategy would effectively exclude individuals who do not necessarily connect their sexual practices with a specific sexual or social identity.

## Background

What happens when normative ‘global’ discourses of rights and individuated sexual identity confront the messiness of ‘local’ realities? What tensions and fault-lines emerge when rights claims couched in the language of identity politics are exercised in contexts in which the relationship between sexual and social identities is neither clear-cut nor unidirectional? In this paper, I discuss the implications for a public health and sexual rights activism of such tensionsthrough an analysis of debates over the naming of male-to-male sexualities/desires – the so-called gay-MSM-koti ‘wars’ characteristic of the politics of male sexuality in South Asia [1-5]. I do so within the specific context of a postcolonial nation-state – Bangladesh – with a highly liberalized economy and transnational socio-cultural sphere. Moving away from a binary framework of authentic/inauthentic or relativism/universalism, I use a transnational analysis to track the ways in which both ‘global’ and ‘local’ understandings shape discourses on sexuality, culture and identity. The meanings and use of categories such as gay, koti (‘effeminate’ males, who feel like women inside and are sexually attracted to men) and MSM (men who have sex with men), are profoundly shaped by what I describe as the NGO-isation of cultural space characteristic of Bangladesh as well as the increasingly globalized currency of particular modes of ‘gay’ culture.

I make two distinct but related arguments in this paper. First, as descriptors, globalized identity categories such as MSM (used by public health providers, the state and donors), and gay/lesbian (invoked by human rights activists and transnational NGOs) are too narrow to capture the fluid and highly context-specific ways in which gender and sexually nonconforming persons understand themselves in Bangladesh. To underline this point I use the case of koti and hijra. Hijra is an ‘indigenous’ South Asian category. In the popular imagination, they are either thought of as a third sex, neither man nor woman, or as ‘eunuchs’, shorn of the capacity for sex. Intersex and male-to-female ‘transgender’ persons, such as koti are often categorized as hijra. However, individuals must be inducted into hijra communities, which have specific linguistic and cultural practices.In this paper, I use hijra as a cultural identity rather than a biological one.

Drawing on legal theorist Sonia Katyal’s insights, this paper argues that a simple politics of recognition will be inadequate to the task of promoting health and human rights for all; such a strategy would effectively exclude individuals who do not necessarily connect their sexual practices with a specific sexual or social identity [6]. Katyal argues that the central paradox of global gay rights discourse is that it can be exclusionary. An unyielding focus on individualized identity excludes those who do not fall into neatly demarcated categories. In relation to the globalization of US gay rights legal strategies, Katyal shows that laws based on the protection of sexual orientation impose and require a relationship between identity and conduct that is highly context-specific. She contends that arguments for legal protection on the basis of sexual orientation often collide with rather than incorporate pre-existing social meanings of same-sex sexual activity [6].

Second, class position mediates to a significant degree the reception, appropriation or rejection of transnational categories such as MSM and LGBT. Indeed, the global circuits through which the terms MSM and ‘gay’ travel, although overlapping, are distinctly class-specific. Thus, more affluent youth in Bangladesh who have internet access and knowledge of English can connect with peers in the ‘global gay community’ and the LGBT movement; those from poorer backgrounds, the ‘targets’ of public health providers, find themselves incorporated into an ‘NGO-enabled’ koti/hijra/MSM community. Furthermore, ideologically, the two transnational discourses of MSM and gay currently circulating are in tension with each other, if not mutually exclusive. The tension is reflected in the sometime fraught relations in Bangladesh between service providers to MSM, the people they serve and the ‘gay community’.

## Methods

The material in this paper draws on a larger research report commissioned by the Centre for Gender, Sexuality and HIV/AIDS at James P Grant School of Public Health, BRAC University (JPGSPH) with financial support from the Realising Rights Research Programme Consortium (funded by the UK Department for International Development – DFID). The research project sought to broaden social understandings of sexuality and sexual rights in Bangladesh, drawing on and expanding the parameters set by conventional public health concerns of HIV/AIDS prevention and harm reduction.

Typically, research projects generated by the AIDS epidemic limit themselves to exploring variables that seem obviously related to issues of transmission and behaviour change such as condom use or numbers of partners in a fixed time period. As one critic, John Gagnon observes:

“All of this research has been governed by interest in AIDS, not interest in sexuality. […] The role of sexuality in the wider social life of the respondents is largely unexamined, the studies are not of ways of living in which sexuality is embedded but are of the variables that are related to measuring transmission or responses to interventions. *In these studies sexuality is a problem but it is not problematized.* […] What sex means to men and women in various cultural situations and its importance (or unimportance) to non-sexual aspects of living is rarely addressed in issues of how often, how many, and did you use a condom the last time and on to the next respondent” ([7]. p 50-51, emphasis added).

The rapid survey interview method, typical of this kind of research, is not designed to produce the ‘thick description’ and cultural complexities to which Gagnon refers.

This study sought to problematize sexuality as a topic as well as to situate sexuality and rights more meaningfully within contemporary urban Bangladesh. That is, a fundamental objective was to produce a *contextualized* understanding of sexuality and sexual rights by mapping the relationship between social and sexual identities; and tracking understandings and negotiations of sexuality among mainstream and marginalized groupsas they are shaped by legal, medical and popular discourses. As such, the project was exploratory and modest in scope, with an emphasis onqualitative research tools. The aim was to generate a conceptual and analytical framework around sexuality and rights rather to than to produce an empirical survey of any one population. A study of this kind is one way of revealing the dynamics of processes that are not necessarily visible otherwise. It also points to questions that require further and more detailed investigation.

Dhaka city and its environs provided the location for the study. The experience of living in the city was common to those selected. Divided by class, occupation and sexuality/gender differences, they represented different segments of the urban population. These were:

❖ Public university students – male and female;

❖ Garment workers – male and female; Women constitute over 70% of the labour force in the garment industry, which employs around 3 million workers, most of whom are migrants from rural areas. The availability of jobs in this sector and the highly visible concentration of women in the urban workforce have had a profound impact on gender relations and individual women’s livelihood options. In the context of Bangladesh, garment workers are an especially significant group to track for changes in understandings of female sexuality.

❖ Individuals from marginalized communities (koti/hijra, women and men in same-sex relationships, MSM);

These three sets of respondents were between the ages of 18 and 28, with a few outliers.

Staff, activists and researchers from organizations working on sexuality, sexual health, reproductive rights and Violence Against Women issues, as well as members of the media, constituted the fourth set consulted for the study.

The complex nature of the subject matter called for the use of ethnographic and open-ended research tools. Formal focus group discussions (FGDs) and semi-structured in-depth interviews, informal conversations and on-site observation were primary tools. Personal narratives, including positive and negative experiences, were also collected. I should note here that informal interaction: “hanging out” in the ethnographic sense, yielded more information than more formal tools of research on many occasions. Conversations over tea and snacks before formal interviews were often more revealing than the taped interview itself.

The research team, comprised of the author and two research associates, carried out four focus group discussions each with the first three sets of respondents and in-depth interviews with specific individuals from these sets. Table 1 and Table 2 show the number of FGDs and interviews carried out in each group. The team carried out in-depth interviews with seven professionals belonging to the fourth set.

**Table 1:** Focus group discussions conducted

2 FGDs with male university students
2 FGDs with female university students (including one group of veiled women)


2 FGDs with female garment workers
2 FGDs with male garment workers



1 FGD with members of BoB (Boys Only Bangladesh)
1 FGD with MSM
1 FGD with hijra/koti
All day discussion with 2 self-identified shomopremi nari (women who love women)

**Table 2:** Individual structured interviews conducted

2 self-identified gay members of BoB
1 self-identified bisexual member of BoB
1 self-identified lesbian (brief interview)
2 hijra
1 MSM


2 female garment workers
2 male garment workers
2 male students

It proved straightforward to organise focus group discussions and individual interviews with university students and garments workers. In the case of same-sex desiring women, it proved to be impossible to carry out anything like a group discussion. Locating respondents was difficult enough; considerations of confidentiality and logistics precluded gathering in the same place at the same time. We interviewed two women who identified themselves as ‘women who love other women’ (shomopremi), and one self-identified lesbian. Self-identified gay men were less wary but initially insisted that a formal group discussion be carried out by one of three individuals already interviewed for the study. The research team did not explicitly include sex workers as members of a marginalized sexual community. However, the hijra we met through NGOs were all sex workers. The person through whom we made contact with other hijra had just been elected president of the Sex Workers Network of Bangladesh. Most sex workers are not hijra but many hijra have turned to sex work in the face of enduring social and economic discrimination. Several of the MSM turned out also to be sex workers.

In addition to formal interviews and group discussion, research team members held numerous informal conversations with hijra respondents and members of Boys Only Bangladesh (BoB). Some of these interactions took place over lunches that were planned ahead but many were also spontaneous and the indirect outcome of the research process. The institutional profile of the Center for Gender, Sexuality and HIV at JPGSPH where the study was housed provided a “respectable” and neutral platform for discussions on non-normative sexuality and rights. The Center became a safe space of sorts, a place where marginalized individuals could converge. As a result of this study and other activities carried out by the Center, individual respondents would often show up for other kinds of assistance or simply to chat.

These encounters provided critical source of information, not reflected in the number of formal interviews and group discussions carried out by the research team.

The complex nature of the subject matter called for the use, additionally, of processual and open-ended research tools. Accordingly, qualitatively different relationships with respondents developed than would have been possible otherwise. The flexible nature of the research process bridged the gap between what respondents themselves saw as significant for their lives and the objectives of the researchers. A hijra respondent noted the difference between interviews she gave for standard surveys and the open-ended conversations facilitated by this study. The former required specific data, and usually did not brook straying from the research topic. She said that at times, this inadvertently made her feel like a token hijra or a non-person. She preferred the more expansive conversations she had with the research team because, in her words:

“It’s not always possible to say everything in all fora, everywhere. To be able to talk about what’s in our hearts, to talk about our bodies, that’s what we want. It’s only because of not being able to say these things that we’ve been forced to leave our households, our families and society.”

Formally, the two-month research period began in August 2008. Informally, locating individuals willing to be interviewed (a procedure that depended on personal connections of the research team and building trust over time) sometimes took much longer. The MSM/koti respondents were easier to locate, through NGOs working on male sexual health. The discussion and analysis in this paper is limited to gender and sexually non-conforming people associated with the service provision activities of public health NGOs.

Strikingly, some groups were more eager than others to tell their stories, and were remarkably frank in their narratives. This included the more visible or openly stigmatized populations such as hijra, as well as MSM and some gay men. Others were much less forthcoming. Women garment workers – the object of much development research -- were particularly reticent to speak on matters related to sexuality. This came as no surprise given that the reputation and social respectability of these working class women are constantly “in question” because of their work [8,9]. Obtaining sensitive and private information is possible of course but the limitations of a short-term study were not always conducive to discussions on sexuality of the kind we desired.

Thus, in terms of access, regardless of sexual orientation and identity, conventional gender lines proved to be well entrenched. We obtained richer material from male gender and sexually non-conforming populations; hence the stronger focus on male sexualities in this article.

As stated above, the objective was to produce an analytical and conceptual framework that can be used to understand sexuality and rights in general in Bangladesh, not to provide detailed descriptions of any one population. As such, the study was indicative of trends in the capital city, not nationally representative.

## Results

The study found, first, that procreative heterosexual marriage is central to the regulation and experience of sexuality across the board*. *For all sets of respondents, marriage was the fundamental axis around which other considerations related to sexuality turned. This reflects the centrality of marriage to the social construction of sexuality and identity in Bangladeshi society.

Second, a range of sexual behaviours is nevertheless sanctioned or tolerated or ignored, as long as such activities remain hidden from the public gaze and they do not disrupt the ideal of procreative heterosexual marriage that is more or less mandatory for men and women. Indeed, it has been argued that sexual taboos in society are not so much about sexuality as about bringing into the public domain matters that should remain private [‘unpublished data’ by Chowdhury A: Exploring Bengali sexuality: Tentative Observations, unpublished paper circulated at the *First International Sexuality Conference*, James P Grant School of Public Health, BRAC University; 2008] [10]. In other words, certain sexual practices may be tolerated when they are hidden from view. Once they are made public, they disrupt the visible order of things, and so enter the realm of the forbidden or taboo.

Third, experiences are highly gendered. Across class and educational lines, regardless of sexual and gender preferences, men possess greater mobility and freedom while women negotiate a more limited set of alternatives. Men have greater ‘freedom’ to act on their sexual or gender preferences -- as long as social and familial obligations are met through marriage, and all acts are hidden from public view. Thus, it is possible for men to lead dual lives. A married man may be able to go ‘cruising’ at night or be in a long-term relationship with another man without encountering too much scrutiny. For some men, marriage to a woman offers a way to ’pass’ as straight in society; for others, it may be desirable for other reasons. Male-male sexual relationships are tolerated under the surface but harshly punished once discovered.

Fourth, a heterosexual/homosexual binary understanding of sexuality cannot be mapped unproblematically onto ground realities. Who is ‘straight’ and who is not is by no means self-evident. The reality is very messy and inconsistent. By the same token, sexual practices and acts do not necessarily come with a social identity. That is, a consistent relationship between sexual practices and social identity cannot necessarily be assumed. An individual’s sexual desires and social desires can go in different directions. Sexual identities are not only fluid and overlapping but they are contextual and contingent. This makes them difficult to pin down.

Fifth, the focus within public health research on HIV/AIDs has had contradictory effects. On the one hand, it has medicalized sexual identities so that hijra or sex workers are associated with disease, thereby incurring the risk of reducing their subjectivity to their stigmatized bodies. Too much of a focus on the dangers of HIV takes away attention from other equally legitimate concerns. Other medical issues, as well as socio-economic considerations, tend to be sidelined. According to respondents, a disproportionate amount of available funding goes to AIDS prevention, sidelining other health and social concerns of sexually marginalized populations. On the other hand, HIV prevention efforts have also opened up many spaces for visibility, mobilization and resources. Indeed, NGO and government intervention programmes, international discourses, as well as the associated research and writing processes, inform and institutionalize sexual identities in important and unpredictable ways [11-14].

Finally, the internet has been critical in connecting isolated individuals and linking them to like-minded others. Gay, lesbian and other same-sex desiring individuals have overridden the unavailability of physical spaces to meet by forming numerous cyber communities. However, this access is class-specific since literacy in English, access to and familiarity with the internet are critical factors in enabling online communications.

### Gays, MSMs and the politics of naming

In Bangladesh, as in the rest of South Asia, the most obvious distinction between those who call themselves gay and those who are defined under the umbrella term MSM is that of class. MSM is a developmental category generated by public health officials and deployed by NGOs as part of outreach services catering to indigent male populations considered to be “at high risk” for HIV infection. Almost by definition, those who are MSM are poor and marginalized socially as well as sexually. Cooks, rickshaw pullers, day labourers and factory workers constitute the rank and file of the MSM population. There are the occasional musicians and dancers as well. They are a disparate group tied together by poverty, deprivation and social exclusion rather than a unified sense of identity. Some are married and lead dual lives. Some stop having sex with men after they get married. When they approach health providers, most do not have a name or identity in mind. This reflects the original formulation of MSM, which sought to accommodate a range of behaviours and identities, including practices that are not culturally or linguistically marked. Proponents argue that MSM is a mechanical and descriptive term with the benefit of being culturally neutral; it was not meant to be constitutive of a separate identity or political community.

In contrast, self-identified gay men tend to be well-educated, have access to the internet, be fluent in English and possess other markers of cosmopolitanism, including close connections to global gay culture. From middle and upper class backgrounds, gay men tend to have more contact with the ‘west’ especially through the internet and travel. Many are members of an online gay forum, BoB, which holds regular social events where gay men can socialize with one another. Entry to these events requires buying relatively expensive tickets, usually out of the reach of the MSM category.

Most gay men in Bangladesh are highly critical of the term MSM, which they consider to be derogatory and apolitical, effectively reducing men’s actions to their sexual urges. In the words of a coordinator of BoB:

“This is not any category! It’s totally a derogatory term. […] even in the workshop in Nepal, we made BoB’s position very clear about this, that we don’t approve of the term ‘MSM’. *Call us gay*, *call us fag*, *but not MSM!* Our logic here is quite simple. ‘Male having sex with male’, [doesn’t make any sense], *it’s not just about sex*. We consider this as an orientation, as an identity. And when you consider this to be an identity, there are so many issues that are interrelated; rights, psychological involvement. And of course *we just don’t go for sex with a guy*, *it’s not like a quick shag! It’s more like a relationship*, *where there is love and romance.* And you don’t call a straight male MSW! So why MSM! I guess it is the government’s strategy to address the health issue without bringing in the rights-based issues. So we have never approved of this term.” (emphasis mine)

He adds:

“This is another reason why BoB has not registered as an organization. We will either register if we can register under LGBTI or Queer identity, or we won’t. The other day we were having this meeting with a Norwegian LGBT organization and they were asking us why we don’t register and get funding to do some more work and we told them that BoB will stay as they are and we don’t want to register. And of course we will not register using the name MSM. And since we don’t work directly with HIV/AIDS, there’s no point to register [using that platform]. Maybe we are being naïve or stupid, given the current situation. But that’s how [we want things] to develop.”

BoB’s coordinator articulately and passionately lays out his reasons for resisting the MSM label. His concerns that gay men’s subjectivities are in danger of being reduced to their sex acts and of the potentially depoliticizing effects of HIV outreach programmes are important. Outreach work based exclusively on HIV prevention, especially when glossed as programmes on men’s health, can easily bypass questions of rights and identities. Significantly, critiques of MSM also incorporate critiques of western-imposed and essentializing development discourse.

To those who take the considerable social and physical risks of identifying as gay, MSM signifies a pre-emptive political capitulation to conservative forces, including governments and donors, and a refusal to acknowledge the reality of homosexuality globally. An oft-repeated story is that no organization that carries out HIV-prevention programmes with MSM will allow its employees to actively embrace a gay identity. In sum, the gay community tends to discount organizations that work only with the MSM population, although this attitude has shifted over the last two years.

Such critiques of MSM can themselves slip into a universalising framework, one in which identifying as gay indicates participation in an emancipatory and modern project. By this logic, MSM have yet to arrive at an emancipatory consciousness. There are serious intellectual and political pitfalls of this universalizing tendency. The assumption, not always implicit, is that as a category gay is superior to all others and as such exemplifies the highest stage of the development of sexual identity. Further, sexual and social identities are assumed to converge. By this logic, same-sex desiring men who do not identify as gay are either in denial or at a ‘lower’ stage of development. This line of thinking does not leave any space for individuals who do not view their sexual practices through the grid of identity. The implicit ranking of differences between MSM and gay can also generate tensions that get in the way of service provision, political solidarity and organizing. Scholars have also noted that this evolutionary framework reinforces notions of western superiority and non-western and/or Islamic backwardness [15-18].

Discrepancies between MSM and gay worldviews are better understood in terms of the distinct needs and interests that arise from differing class and social positions. Class divides in small but significant ways, undermining certain aspects of the supposed universality of gay experience. It is worth noting, for instance, that most poor men, regardless of how they conceptualize their sexual and gender identities, do not have access to medical or other services except through organizations that work with MSM. Service delivery is actually quite critical to these men’s lives.

In their everyday lives, MSM/gay subjectivities and experiences emerge in small but subtle ways. For instance, on the emergence of gay identity in South Africa, Donald Donham notes how going to the library or searching the internet for ‘others like me’ is a central factor in coming out for many gay men across societies [19].

Like gay men, MSM interviewed for the study talked of finding others like themselves. Unlike middle class individuals, they did so not through books or the internet, neither of which they had access to, but through the organization for which they worked. A senior field staff member of an NGO said,

“I came here and found others like me. That’s what I liked about this place - what I can’t tell my parents or brothers and sisters, I can share with my friends here, because they are my fellow travelers.”

It may be that the availability of a transnational gay identity so clearly mediated by class, can itself be homogenizing and limiting. A member of BoB who was an upcoming young professional said that unlike hijra, he would never be able to cross-dress even though he wanted to. When prodded, he mused,

“Maybe I’m not gay, maybe I would be transgender in a different world.”

### Cultural uses of the MSM label

In India, some activists argue that “the emergence of the category of ‘MSM’ is an assertion of racial/cultural/social difference by South Asian gays, consciously interrogating universalized notions of ‘gay’-ness in this case” [20] [p xxiii]. Notably, interrogation of this kind has been absent in Bangladesh. Regardless, if the objective of the MSM tag in public health discourse was to avoid engaging in frequently intractable political battles over the cultural translatability of terms such as gay/homosexual, then it can be said to be successful. The vagueness of the term allows it to avoid imposing fixed and sometimes external cultural meanings to same-sex practices in different contexts and locations. Such a classification covers practices that do not map onto existing or emergent identities. Because it provides a tag for persons who refuse a specific sexual identity because they do not know or perhaps do not care, MSM is a heuristic device above all.

Observations from the study suggest that most of the men classed together as MSM would be labeled transgender in another context, though not hijra. Most have learned to call themselves kotis (effeminate men, or men who feel like women inside). Gender role, not sexual orientation, is at issue [21-23]. If gay identity depends upon the gender of one’s sexual partner, then these MSM/koti are gay. But kotis define themselves not by the gender of their sexual partners but by the gender roles with which they identify:

“I used to feel girly all the time and kept feeling attraction for boys. I used to want to sit at home like a girl, not go out and play cricket.”

“So what if I’m a man, inside/in my heart I’m a girl. I wanted so much to have big breasts but I couldn’t. I took many, many pills. I knew they were going to be bad for my health but I couldn’t stop.”

Kotis or effeminate males often have long-term relationships with panthis. These are men who seek them out for ‘fun’, ‘sexual release’ or genuine affection. While all the kotis interviewed in the study longed for these relationships to last, the active male partners move from the public world of work and family to the hidden world of kotis with their hegemonic masculinities seemingly intact. Whether these active sexual partners have emotional or identity investments in these relationships varies from one individual to another. Invariably these men enter heterosexual marriages, either out of social pressure or out of a desire for a different and more secure future. Sometimes they are comfortable leading dual lives, although the less affluent are always vulnerable to socio-economic discrimination and violence [24-26].

Significantly, koti is not necessarily a pure “pre-NGO” cultural category. It is through NGOs that work with MSM that many people encounter and embrace the purportedly “indigenous” term koti. This became clear during a focus group discussion at an MSM organization:

“*there are so many new kotis*, *they don’t even know what a koti or panthi is*. I was like that too. For those who come in new, it’s a problem [naming].”

During a group discussion at an NGO serving the MSM population, everyone in the room was asked how they identified themselves. One person introduced himself as a ‘MP’. He explained he was a ‘Male Prostitute’, the classificatory term used for him at the NGO, and the answer he assumed that the researchers sought. The apparent ease with which he used the term gives some insight into the temporality of identity: MP happened to be a category this individual adopted in the context of NGO-related activity.

The group noted that being a koti carried special health risks (such as greater risk of ruptured anus, higher susceptibility to STIs and HIV) and that this should be highlighted during counseling sessions. But they also noted that,‘every individual has a different level of risk’. They did not reproduce universalistic categorizations of risk levels and their approach to the term MSM appears to be one of pragmatism.

“Most people know us as koti. We don’t call ourselves MSM outside, why should we, how many people know what it is? We hear these terms in seminars. Government offices have picked up this vocabulary. Moreover, *MSM doesn’t give the specificity of what it means to be a koti or the passive partner*, therefore most prefer the term koti to MSM.”

“MSM implies an active role but we kotis, we do a passive role, so we are happier when we’re referred to as koti.”

When pushed, one of the men present said,

“Maybe it’s as you said and it’s because of this organization. There’s still so much I don’t know. *I’m koti because someone brought me to this organization*, where I saw there were other people like me, using the language of hijra – of koti, gotiya (female friend) and panthi (those who take on the active role, of penetrator rather than the one who is penetrated, in sex).”

These categories are thus very fluid and shifting. There are substantial linguistic and other overlaps between hijra and kotis; many of the terms they use are part of the secret language or argot used by hijra. This blurring of lines is frequently contested by kotis themselves:

“Some people lump us with hijra, they don’t understand, because we have some similarities with hijra. In some domestic spaces, we are made into and kept as hijra.”

The instability of hijra identity was brought home in the course of a group discussion with hijra sex workers. Abanti, a striking beautiful woman who carried herself with unusual grace, narrated the story of her social transformation from her male self to a female identity. What could have been a triumphal account of liberation from the shackles of having to live in a man’s body turned into a story of loss and mourning for her male self. Abanti said she often thought of the male self she had had to sacrifice in order to become Abanti.

What are theorists or activists to make of this story? That selves are multiple, inconsistent and shifting? An emancipatory narrative of the individual coming into being cannot possibly capture the ambivalence, pain and longing to which Abanti alludes [27].

It is instructive to get a sense of how kotis/MSM conceptualize the term gay, and the differences between them and gay men. There are a range of views, although the primary distinction is around gender roles in the sex act:

“As far as I’ve heard, gay is someone to whom a man gives, and then when it’s done, he gives to the man. But with koti-panthi sex, it’s not like that. With us, it’s that the panthi will continue to give and the koti will continue to take.”

This person went on distinguish the sex roles kotis may take on during commercial sex work, from the roles that are the outcome of desire. Thus,

“*We may play different roles at times but that kind of desire isn’t there*. If someone pays, and a koti wants to take from another koti, then we have no choice. We might not like or enjoy, but we do it.”

But he acknowledges the fluidity of sexual preferences and desires, and so of gender identity:

“Of course, occasionally a koti might be ‘diverted’ (uses English word) and will want to take (be dominant) rather than give (passive).”

Gay is a word with which these kotis are all familiar, and which some try to contextualize:

“Gay is someone who is do-paratha, that is, someone who plays both roles (someone who takes on both passive - the penetrated - and active - the penetrator -sex roles).”

“In our community, they’re called do-paratha. We don’t see any difference. The things we do, they do as well. The difference is that we do more of the female roles. Koti-panthis have relationships with them too. Some of them are active, some passive. That’s gay.”

“Their needs and desires are the same. Actually it’s not at all clear. It seems to me that those of us who are koti are not clear, sexuality is complex, not clear. They don’t know themselves who they are, what they want. But even they will act like gays because their conception of sexuality isn’t clear. *They just know what they like*. *Because of this lack of clarity*, *if the organization says we’re MSM*, *then we’re MSM*, *if they say we’re koti*, *then we’re koti. But there are gays who just think of themselves as gay.*”

The speaker conflates fluidity with a lack of clarity and betrays a desire for the certainty of identity gay men supposedly have. At this discussion, someone else immediately counteracts the uncertainty expressed above:

“Actually, we *are* clear about these things. Gays are less effeminate. We know who will give and who will take. But among gays, each one has such a huge appetite, you can’t tell who will give (be penetrated) and who will take (penetrate).”

These group discussions show how local realities and complexities make the term MSM, which includes kotis, panthis and hijra but excludes gays, very problematic as a way of categorizing sexual roles and behaviours.

### Intersections of global and local discourses on rights and sexualities

The convergence of increased donor interest in sexual rights, the availability of new communications technology and connections to transnational gay and lesbian culture are critical factors in enabling new voices on sexuality and rights to emerge in Bangladesh. The story of the moderator of a platform for self-identified gay men that began as an exclusively online forum, is instructive:

“I had lived abroad since childhood. I’ve had access to the internet since 1996, so I was familiar with the terms and the issues. I was in touch with people in other parts of the world who also belonged to the community so I did not suffer any mental stress regarding my identity. When I returned in 2001, I had no idea there was a community here. I met a guy online at BD chat (a closed listserve for gay men in Bangladesh), it was the happening site at the time. He introduced me to the [online forum.] That’s how I got involved.”

Notably, for him, ‘the [gay] community’ has unambiguous borders and is international by definition. He sees himself as part of a wider global gay community. It is on the internet that the moderator came across a notice for a workshop on sexual diversity and rights being organized by the Blue Diamond Society in Nepal. Participants at the workshop founded the Nepal-based South Asian Human Rights Commission for Marginalized Sexualities and Genders (SAHRCMSG) in September 2008. In February 2009, the Bangladeshi organization held a workshop on sexuality diversity and coalition building with the support of a Norwegian NGO whose members BoB’s moderator had met in Nepal. The gathering was historic in that it brought together for the first time self-identified gays and lesbians (members of the online group) with MSM, hijra and members of a same-sex loving women’s organization. Some of the core debates at the meeting, sometimes rancorous, turned on naming practices of the various groups present.

It should be noted here that BoB is not a foreign-funded initiative but one that has benefited from an influx of international funds and resources in the last two years, partly as a result of the enterprising attitude of its moderators. Indeed, the name Boys Only Bangladesh indicates a desire to domesticate and nationalize what it means to be gay.

### NGOs and the production of sexual identities

As the various accounts above indicate, the social meanings of same-sex sexual activities cannot be contained by any one category. The movement from linguistically and culturally unmarked to official marked categories and identities rarely accommodates the extraordinary fluidity in individual practices, preferences and self-expression [28-31].

The language of NGO interventions along with transnational gay cultural formations provides major structuring discourses and available identities for males. In conjunction with local meanings and vocabulary and structural factors such as class position, it provides the parameters for individual identity formation. Working class males who have sex with other males, find that koti/panthi and hijra are the only cultural categories available to them. Unless they enter into a hijra community, most use koti for personal identity, MSM for seeking services.

Complications arise because of the structure of funding, government constraints and the imperatives of transnational gay politics. The government does not recognize the category ‘gay’; all funds channeled through the NGO Affairs Bureau are directed at hijra and MSM. This is an integral aspect of NGO governmentality in Bangladesh.

In their current structure, NGOs are set up to provide services to specific communities (those ‘at risk’). But in order to do so, they have to define the parameters of the said community. Individuals must enter as members of the community in order to use NGO services, making the option of exit or experimentation with identity less feasible. The much-circulated story of a long-term employee of an MSM organization fired for insisting on his gay identity can be understood in this light. Through his actions, this employee of an organization that worked on MSM clearly undermined the claim that MSM and gay are entirely separate and unrelated categories, therefore undermining the interests of the NGO.

NGOs provide one of the few avenues of respectable livelihood for sexually marginalized communities. But that also traps the individual within the classificatory categories of the NGO concerned – this is a question of funding and global connections and classifications. Competition around funding has intensified a process that can be termed the NGO-isation of identities. At a recent meeting at the School of Public Health, a hijra activist said, only half-jokingly:

“If I go to NGO X, I call myself Hijra, at NGO Y, I’m Koti, and at NGO Z, I present myself as MSM.”

For the subjects of this study, the overarching context for sexual subject formation appears to be public health NGOs and transnational gay culture. Online fora for gay men and NGOs that provide male sexual health services are institutional sites in which new transnational subjectivities are being produced. They act as support groups of a sort. The crucial distinction is not between externally imposed versus ‘authentic’ indigenous subjects. In this analysis, it is the intra-national– the difference in class – which generates two quite distinct subject-positions and locations.

## **Conclusion: sexual rights activism beyond the identity conundrum**

The liberal notion of a bounded and autonomous rights-bearing individual has come under considerable critique from political theorists and postcolonial scholars in recent years. The formal language of rights – which assumes the abstraction of a self-contained individual, is often conceptually and analytically removed from the messiness of lived experiences of historically and culturally located subjects. In this paper, I have tried to address this gap by revisiting the politics of naming in relation to the MSM/Gay debate in Bangladesh.

The analysis in this paper suggests that first, we cannot assume an equation between sexual conduct and sexual identity in Bangladesh and second, along with fluidity, many men exhibit indeterminacy in relation to sexual identities. Further, homogenizing categories and forms of discourse – such as MSM and gay – that circulate globally invariably alter social relations and meanings on the ground. That is, the pre-existing social meanings of same-sex sexual activities are themselves changed through attempts at definitional fixity required by both social movements and NGO interventions.

That sexualities and sexual identities are fluid, multiple and overlapping is more or less a commonplace. What such fluidity entails for individuals seeking rights as citizens of a state or as members of a community is not self-evident [32]. If sexual identities are not only fluid but also unstable, how do we contextualize rights or proceed with sexual rights activism? Delinking rights from individual identities, and turning our gaze to broader structures of power and inequality, would be one way to reframe the debate.

Normative sexualities are intrinsically linked to ideologies of domination and inequality; sexual inequalities are embedded in a host of political and social inequalities. For instance, when MSM and hijra are refused medical treatment or harassed by police on the streets, it is as much their class standing as their non-normative sexuality that works against them. Sex workers and poor women, especially if they are single, routinely experience harassment that is both class-based and sexualized. Sexual and gender non- conforming individuals – of whatever class – are considered threats to the social order if they visibly refuse hetero-normative norms as manifest most notably through marriage. This suggests that locating sexual rights activism within broader struggles for social justice, including struggles for the right to health and wellbeing for all, regardless of sexuality, would be a place to start.

## List of abbreviations

BoB: Boys only Bangladesh; JPGSPH: James P Grant School of Public Health; LGBT: Lesbian, Gay, Bisexual & Transgender; MSM: Men who have Sex with Men; NGO: Non-governmental Organisation; SAHRCMSG: South Asian Human Rights Commission for Marginalized Sexualities and Genders.

## Competing interests

The author declares she has no competing interests.
